# Pain Resilience and Coping Behaviors in Individuals in a Collectivist Social Context

**DOI:** 10.3390/healthcare12191979

**Published:** 2024-10-04

**Authors:** Ling-Jun Liu, Hsiu-Ling Peng, Wan-Ping Liang, Edward Meng-Hua Lin

**Affiliations:** 1 Department of Anesthesiology, Changhua Christian Hospital, Changhua 500, Taiwan; lingjun.liu1203@gmail.com; 2 Department of Statistics, Tunghai University, Taichung 40704, Taiwan; 3 Department of Psychology, Chung Shan Medical University, Taichung 40201, Taiwan; 4 Department of Pastoral Care, Changhua Christian Hospital, Changhua 500, Taiwan

**Keywords:** resilience, chronic pain, qualitative, spirituality, collectivism

## Abstract

Background/Objectives: Pain resilience (PR) may be associated with different coping skills, resulting in differences in pain outcomes. This study aimed to understand the role of PR in dictating the choice of coping methods. Methods: This study completed a preliminary validation of the Mandarin Chinese version of the Pain Resilience Scale (PRS-C) with online survey data (n = 46). Further, we conducted interviews with individuals with chronic low back pain (n = 24). Results: The PRS-C psychometric properties were assessed using a confirmatory factor analysis. The interviews explored pain history, treatment experiences, and coping strategies and were analyzed thematically. The validated PRS-C (10 items) demonstrated satisfactory psychometric properties. The interview results showed that participants who scored lower were more likely to adopt disengagement rather than engagement coping strategies. Qualitative data revealed three themes explaining why and how participants in this collectivist social framework chose their coping methods. Conclusions: The findings suggest that while participants tried to understand their pain and treatment experiences, the cognitive appraisal construct in PR influenced some of the coping experiences. However, deeply influenced by Confucianism and Buddhism, participants also expressed factors beyond the scope of individual fortitudes, such as the relationship with a higher power that significantly influenced their coping behaviors.

## 1. Introduction

Pain resilience (PR) describes the positive psychological functioning associated with physical and emotional improvement in individuals experiencing pain [1]. PR is strongly associated with general resilience; however, it specifically measures the ability to recover from pain-related experiences. The two factors that contribute to PR are Cognitive/Affective Positivity and Behavioral Perseverance. The former refers to the perceived capacity to regulate emotion and cognition, while the latter refers to behavioral or motivational strengths under intense or persistent pain. The Pain Resilience Scale (PRS) was developed to assess the level of PR and has been validated in undergraduate participants [1], individuals with chronic pain [2], patients with temporomandibular pain disorders [3], individuals with HIV and pain [4], and Chinese individuals with musculoskeletal pain [5]. These studies consistently found that the PRS was positively correlated with general resilience and negatively correlated with indicators of vulnerability, such as depression or fear of movement.

In the original article, the authors suggested that people with high PR developed more adaptive coping skills, leading to better pain outcomes [1]. Typical pain outcomes mentioned in previous studies included quality of life, intensity of pain, drug use, or other daily functioning [2,3,4,6]. Higher PR was found to be associated with a better quality of life, a reduced probability of drug use (opioids and non-opioid prescription medications), and a reduced probability of unemployment [2,6]. However, there is a lack of discussion on why PR is associated with better pain outcomes. How did this positive psychological functioning result in positive behavioral or functional daily performances? Perhaps the missing puzzle piece in the associative findings between PR and outcomes is coping skills, as suggested by the original authors of the PRS [1].

Coping skills or coping experiences can be heterogeneous, and the stability of responses can vary with situational challenges [7]. Even with a comprehensive questionnaire on pain coping, e.g., [8,9,10], it remains difficult to understand why a person adopts certain coping strategies and the role of PR in dictating the choice of coping methods. Another factor that may lead to differences in pain coping may be the personal identification with individualism or collectivism. A previous study conducted in the United States found that participants who identified more with individualism were more prone to seeking formal treatments for pain and engage in more active coping strategies. The same phenomena were not observed in participants who identified more with collectivism [11]. However, the authors emphasized that the study was completed in an individualistically oriented area.

Thus, this study has three objectives. First, we aimed to complete the validity study of the Chinese translation of the PRS (PRS-C) in an area that is collectivist-oriented. Past research indicated that despite the rapid economic development in Taiwan since the early 1970s, people in this area still embrace Chinese collectivism in general [12]. Individuals who identified with collectivism tended to suppress personal desires to conform to social expectations or to maintain interpersonal harmony [12,13]. The PRS-C should demonstrate appropriate psychometric properties. The second objective of this study is to understand the association between PR and coping experiences. We hypothesize that PR should play a role in dictating coping strategies in our sample. To be more specific, individuals with higher PR should display different coping styles from individuals with lower PR. The third purpose of this study is to provide a detailed qualitative narrative about PR and coping strategies in individuals living in a collectivist-oriented area. Until now, studies validating the PRS in different languages or in different pain groups have been completed with questionnaires (i.e., [2,3,4,5]). This study aims to gain insight into the topic by interviewing individuals with chronic pain who live in this collectivist society. We hypothesize that pain coping behaviors in the current sample should also demonstrate conformity to collectivism social norms.

## 2. Materials and Methods

This study was registered at ClinicalTrials.gov (registry number NCT05148364). This is a mixed-method study that includes the quantitative study of scale validity and qualitative interviews. Qualitative narrative data were collected to gain insight to the topic of interest [14]. These data can serve as complementary to the quantitative method to provide deeper understanding of human behaviors, even in the study of pain (e.g., [15]). This study was conducted in two stages: first, the validation of the PRS-C in patients with chronic low back pain, and second, qualitative interviews of patients with chronic low back pain.

### 2.1. Participants

The enrollment criteria for participants in the scale validation and participants in the qualitative interview shared several elements: (a) persistent low back pain for at least three months prior to study, (b) able to communicate in Mandarin Chinese, and (c) over the age of 20 years old.

### 2.2. Sample Size Determination

We determined the sample size for the validation based on the volume of the target population in this medical center. The average number of clinical visits for all types of pain to this pain clinic was 400–500 per month. Considering the prevalence of low back pain in this area [16,17], the target population was estimated to be 64–165 individuals. The sample size for the qualitative interview was based on past studies, with criteria detailed in Section 2.6.

### 2.3. Data Availability

This study adhered to the Journal Article Reporting Standards [18]. Raw data and the analysis code for the validation are available as Supplementary Materials. Data were analyzed using R version 4.3.1 [19] with the following packages: readxl (Wickham & Bryan, 2023, lubridate [20], psych [21], modelr [22], corrplot [23], moments [24], lavaan [25], semPlot [26], GPArotation [27], psychometric [28], and ggplot2 (Wickham, 2016).

### 2.4. Ethical Approval

The validation of the scale was approved by the institutional review board (IRB) of Changhua Christian Hospital (approval number 181230). The validation study was conducted via an anonymous online survey; therefore, the IRB waived the requirement for written informed consent. The qualitative interviews were conducted on site and approved by the IRB of Changhua Christian Hospital (approval number 211102). Each participant provided written informed consent before participating in the interview.

### 2.5. PRS-C Validation

The validation process was guided by the ISPOR guidelines [29]. Permission to validate the PRS-C was obtained from the original authors of the PRS. The scale was translated into Mandarin Chinese by a clinician and then back-translated by another clinician. Subsequently, the research team agreed that the translated version is appropriate. We recruited participants from a pain clinic and through online advertisements. Participants were adults (>20 years) who had chronic low back pain for at least 3 months prior to participating and were able to read traditional Chinese characters. Individuals who passed the screening test completed the following questionnaires: the PRS-C (14 items), the World Health Organization Quality of Life Brief Version (WHOQOL-BREF, 28 items [30,31], the Patient Health Questionnaire (PHQ-9, 9 items) ([32,33], the Pain Catastrophizing Scale (PCS, 13 items) (Refs. [34,35], and the Brief Resilience Scale (BRS, 6 items) (Refs. [36,37]. The WHOQOL-BREF, PHQ-9, PCS, and BRS have been translated and validated well in the Chinese-speaking population. For more details on the psychometric properties of each scale, please refer to the studies cited (please refer to Refs. [31,33,34,37]).

An exploratory factor analysis was first performed to extract latent variables. Values between 0.7 and 0.8 in a Kaiser–Meyer–Olkin (KMO) test for sample adequacy indicate adequate sampling of variables for a later factor analysis, and values greater than 0.8 indicate good sampling [38]. Model fitness was then examined using confirmatory factor analysis. The model fitness indices included the Chi-square test (χ2), root mean square error of approximation (RMSEA), standardized root mean square residual (SRMR), Tucker–Lewis Index (TLI), and comparative fit index (CFI). The χ2 test is an evaluation of the discrepancy between the sample and the fitted covariances matrices [39]. An insignificant result on a χ2 test indicates good model fitness, but this indicator is likely to be rejected when the sample size is large or when the multivariate distribution does not conform to normality. Therefore, other model fit indicators should be taken into consideration. The cutoff values for each index were as follows: RMSEA < 0.06, SRMR < 0.08, and TLI and CFI > 0.95, according to Hu and Bentler [39]. Indicators of model parsimony, such as the Akaike information criterion (AIC) and Bayesian information criterion (BIC), have also been reported [40,41]. Each item was required to have a factor loading of 0.4 on the primary factor, or it is loaded twice as strong on one factor than on another factor [42,43]. If an item was cross-loaded, there had to be a difference of at least 0.2 between the loadings on the primary and the alternative factor [43].

We calculated internal consistency (i.e., Cronbach’s α) to measure reliability. We also reported construct validity (assessed by convergent validity) and external validity. For convergent validity, we calculated the correlation between the PRS-C and other scales commonly used in pain research. The underlying hypothesis is that the PRS-C, a measure of the positive psychological functioning of pain, should be positively correlated with protective factors, such as general resilience (measured with BRS), but negatively correlated with psychological vulnerabilities, such as depression (measured with the PHQ-9) and catastrophizing (measured with the PCS). External validity was evaluated to understand how the PRS-C is associated with a measure indicative of a protective psychosocial condition (measured with the WHOQOL-BREF).

### 2.6. Qualitative Interview

We invited participants from various backgrounds in order to gain an in-depth understanding of the topic. The categorization criteria spanned four psychosocial factors known to determine pain management outcomes: gender [44], religious belief [45], long-term opioid treatment [46,47], and support from social welfare [48]. A combination of these factors results in 16 possible conditions, with a predetermined sample size of 4 participants for each cell, totaling up to 64 participants if data saturation was not reached. However, the actual sample size depended on data saturation, which was the point at which recruitment stopped because additional interviews would no longer provide new information [49]. In this study, the strategy for assessing saturation was based on code and meaning, where the codebook was stabilized and the meanings of codes were captured [50].

Qualitative interviews were conducted in a semi-structured manner by the same interviewer and note-taker. In addition to the interviews, participants filled out the validated PRS-C. All interview questions were pre-planned, covering participants’ pain history, treatment experiences, current lifestyle, employment status, interpretation of each pain attack, and pain coping strategies.

The interviews were recorded for later analysis. Each transcription was coded using the NVivo software 14 [51] by two researchers, one with a background in clinical psychology and the other with a background in spiritual care. All codes were later verified by another researcher with a background in clinical psychology. The transcripts were subjected to thematic analysis as previously outlined [52]. Coping strategies were classified based on the study by Nielsen and Knardahl [53] into engagement coping, disengagement coping, and low coping. This classification loosened up the orthogonal classification that dichotomously distinguished between problem-solving and emotion-focused coping [54]. Engagement coping encompassed problem-solving coping strategies and some forms of emotion-focused coping, such as acceptance and emotional regulation, viewed as proactive methods to manage potential stressors. On the contrary, disengagement coping encompassed maladaptive emotional or behavioral responses. Low coping referred to individuals who scored low on all coping strategies.

In this study, we calculated the number of coping methods mentioned by the participants and categorized the methods under engagement or disengagement. For instance, if a participant mentioned jogging, going to church, and using injectable opioids, they received two points on engagement coping and one point on disengagement coping. Pearson’s correlations were calculated between the amounts of each category of methods and the total score of the PRS-C.

## 3. Results

### 3.1. Validation of PRS-C

A total of 46 participants completed the online survey for the validation of the PRS-C (Table 1). For readers interested in knowing how the current sample rated in the 14-item original scale, the descriptive statistics are also listed in Table 1. The results of the exploratory factor analysis showed that the sampling was adequate (KMO = 0.86). The details of the descriptive statistics of each item are listed in the Supplementary Table. The results of the confirmatory factor analysis indicated that the psychometric properties of the directly translated original PRS were suboptimal. There were many cross-loading items (Table 2), and while the RMSEA was too high, the CFI and TLI were too low (Table 3). Another PRS model proposed by You and Jackson [5] was also evaluated, but it did not exhibit ideal psychometric properties either (Table 3). Model modification should be carried out after discovering an inadequately fitted structural equation model [55]. Items with a high modification index can signify that the item has compromised discriminant validity [56] or that a correlation between an item and the error term of another item exists [57]. Therefore, removing items with a high modification index is an effective way to improve model fitness. Following the approach from a previous study [2], items with high modification indices were progressively removed, eventually leading to the identification of a model with the best psychometric properties upon the removal of items 5, 8, 9, and 14 from the original scale (Table 3). An insignificant χ2 indicated good model fit. All fit indices were ideal, and the RMSEA was within the acceptable range. Factor loadings of each item were above 0.4. Although item 6 showed cross-loadings on two factors, the difference between the loads was over 0.2, justifying its retention in the model.

**Figure 1:**
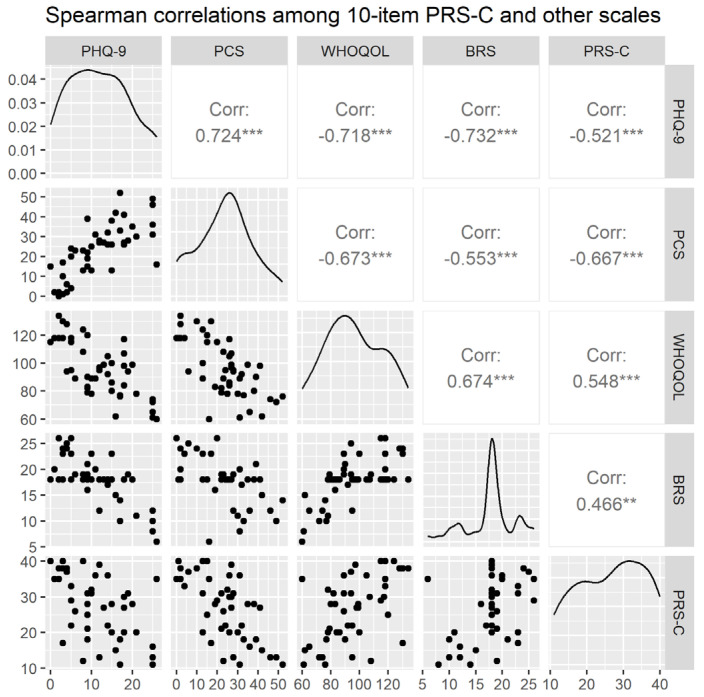
Spearman correlations among 10-item PRS-C and other scales. PRS-C: Chinese version of the Pain Resilience Scale; PHQ-9: Patient Health Questionnaire (9 items); WHOQOL: WHO Quality of Life Taiwan version; BRS: Brief Resilience Scale; PCS: Pain Catastrophizing Scale; ** *p <* 0.01; *** *p <* 0.001.

The total score of the 10-item validated PRS-C exhibited good construct validity and external validity (Figure 1). Spearman correlation should be used when any of the variables is not normally distributed [58]. In the current sample, the distribution of the PHQ-9 is positively skewed (skewness = 0.253). Therefore, Spearman correlation was chosen to analyze the correlations between the PRS-C and other scales. The correlation analysis showed that the PRS-C was positively associated with quality of life and resilience in general but negatively correlated with pain catastrophizing and degree of depression.

### 3.2. Qualitative Interviews

**Figure 2:**
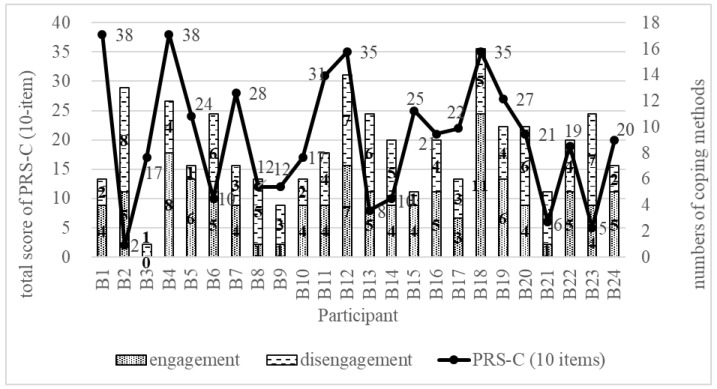
Total scores of PRS-C and counts of coping methods. PRS-C: Chinese version of the Pain Resilience Scale.

Overall, 24 patients with chronic low back pain participated in the interviews, none of whom took part in the online survey for PRS-C validation. Certain patient characteristics were unrepresented (i.e., the empty cells shown in Table 4). Demographic characteristics of the participants are displayed in Table 5. Figure 2 illustrates the number of engagement and disengagement coping methods adopted by each participant.

Compared with the participants not on long-term opioid therapy, most participants using opioids as the main pain control medicine tended to have lower scores on the PRS-C, except for participant B4. In the analysis of the qualitative narratives about coping experiences, code saturation was achieved after 11 interviews, but the recruitment continued until meaning saturation was reached [50]. Data on coping methods repeated across participants with different demographic characteristics, indicating saturation.

The thematic analysis identified three main themes depicting the qualitative aspect of the participants’ coping experiences: “People said”, “The limits of modern medicine”, and “God’s will”.

#### 3.2.1. Theme: “People Said”

Throughout the interviews, many participants talked about how interactions with others influenced their coping methods. Most participants felt stigmatized, and only three participants felt that other people were helpful:

“My daughters said injections can do harm to me, but they cannot understand why I need the injections (of nalbuphine). I have to hide when I use the medication” (participant B12, female, 47 years old, pain > 10 years).

“People around me doubted that I needed these many medicines to alleviate the pain. They say that I am not a cancer patient, why would I need opioids for pain? I constantly have to argue with them over this issue” (participant B13, female, 26 years old, pain for 8 years).

“My kids know about my pain, but they are against me using pain medications or injections. They asked me to get another surgery so I can be ‘normal’ again” (participant B16, male, 49 years old, pain for 13 years).

“I lied to my colleagues that it is medication for diabetes when it is, in fact, nalbuphine. My parents think I am an addict. A girl left me for the medicine I take; she did not believe the medicines were prescribed by doctors” (participant B9, male, 49 years old, pain for >10 years).

“Sometimes my husband would rub my back for me, my pain would relieve a little. Most of the time, I try to hide my pain. I look healthy, and so no one believes that I am in pain” (participant B18, female, 42 years old, pain for >10 years).

#### 3.2.2. Theme: “The Limits of Modern Medicine”

“The effect of drugs wears off more and more quickly. I do not know how to find a cure for my pain” (participant B17, male, 45 years old, pain for 5 years).

“There is only so much pain medication can do. Whenever I think it cannot get any worse, my pain exacerbates. I can only accept it” (participant B5, male, 27 years old, pain for 12 years).

“I feel a little embarrassed to say that as a physical therapist myself, I have low back pain. I know all the nonsurgical options and I have tried most of them, but the pain is still there” (participant B7, male, 40 years old, pain for 6 years).

#### 3.2.3. Theme: “God’s Will”

“I am not a big fan of Fengsui, but my mom is. She asked some psychics and insisted that I drink spell water to cure my pain” (participant B4, female, 42 years old, pain for >10 years).

“There is a temple in my hometown and local people believe in it. My grandma asked God for advice on surgery and received an answer. If it was not for God, the results could be terrible. I feel I was destined to choose that hospital for surgery. I think God helped me” (participant B20, male, 44 years old, pain for 17 years).

“I think God answered my prayers by giving my doctor some ideas about how to treat my pain” (participant B13, female, 26 years old, pain for 8 years).

“I have tried committing suicide, but God has pulled me back again and again” (participant B14, female, 52 years old, pain for 3 years).

The results of the correlational analysis between the amount of coping methods and the PRS-C showed that the PRS-C was positively correlated with the amount of engagement methods (*r* = 0.52) and negatively correlated with the amount of disengagement methods (*r* = –0.34).

## 4. Discussion

This study validated the PRS-C and conducted one-on-one interviews with individuals with chronic low back pain to identify factors influencing their choice of coping strategies that may not be captured through questionnaires. The study design and conduction incorporated researchers with backgrounds in psychology, spiritual care, and statistics, ensuring a multidisciplinary approach that encompasses qualitative and quantitative methods and psychological and spiritual perspectives.

Similar to previous studies, e.g., [2,5], some items were removed from the original scale to obtain better psychometric properties. The first hypothesis of this study was supported. The validated PRS-C showed good psychometric properties. Our findings supported our second hypothesis as well as the original PRS paper’s assertion that PR is associated with coping capabilities [1]. Participants with lower PRS-C scores tended to adopt more disengagement than engagement coping strategies.

Among the disengagement coping strategies, opioid use was a common method repeatedly mentioned by participants. On the contrary, most participants on long-term opioid therapy displayed relatively low PRS-C scores. This finding is consistent with previous findings on opioid use and PR [2]. Moreover, it may complement observations regarding opioid use and physical activity. While opioids can be more effective than placebos in relieving pain, they do not show a superior effect in improving physical activity [59]. PR may help to explain why many individuals on long-term opioid therapy did not engage in more engagement coping strategies after using opioids for pain control. Lower PR is associated with a higher propensity to adopt disengagement coping strategies, such as opioid use. The continuous reinforcement of opioid use, due to its effectiveness in reducing both pain and withdrawal symptoms [60], limits individuals with low PR from seeking strategies that require more perseverance.

Furthermore, our study suggests that cultural and interpersonal factors play a role in determining the coping method one uses. Through interviews, we discovered that the realm of pain coping can be beyond personal control. For example, the theme “People said” found in the qualitative data showed that in addition to personal will, the responses of others can influence personal coping behaviors. The narratives of some of the participants reflected the perception of being stigmatized, especially when they referred to issues surrounding drug use, or the presentation of pain behaviors. This perception of stigmatization was not uncommon in people with persistent pain [15,61]. Most of the respondents have found their way to “hide” their true needs to avoid further judgments or confrontations. This supported our third hypothesis that coping behaviors in the current sample also demonstrated conformity to the social norm of collectivism. The original PRS was validated within an individualistic social framework, but the model did not fit perfectly in studies with Chinese participants, who are part of a collectivist social framework, as seen in You and Jackson [5] and the current study. Endurance, suppression, and catering to other’s needs are highly desirable social behaviors in Confucianism [62,63]. Choosing a socially desirable coping strategy is an essential part of the pain experience of people in this cultural framework. Therefore, items related to endurance, whether these items belonged to the Cognitive/Affective Positivity or Behavioral Perseverance subscale, were preserved. This theme also demonstrated that in a collectivist social framework, family members can try to interfere with or exhibit more judgmental attitudes toward the coping strategies that the participant adopts. This family-oriented pain experience is similar to the findings of a previous qualitative study on ethnic differences in the pain experience of cancer [64].

The theme “God’s will” reflected the individuals’ interpretation of pain experience. In addition to attributing the treatment or recovery from pain to personal efforts, qualitative data in the current study suggest an additional layer of pain coping: participants also ascribed treatment experiences to a higher source of power beyond their control. In Buddhism, pain is often believed to be a punishment for a sin that was caused in the past or even from a past life [63]. Similar to other medical conditions, spirituality was tested in the experience of pain [65]. Individuals tried to make sense of this pain by wondering if they had not done enough to please God, or if they had failed God in some way. This appraisal not only corresponds to the cognitive appraisal construct of PR but also reflects the participants’ trust in a higher power that can support them [66]. The resilience from pain may be partially determined by an individual’s faith in a higher power [67]. This mentality presents a spiritual or religious need that was not presented in the original PRS, potentially contributing to the model’s misfit during the validation of the PRS-C.

PR, or the PRS used to measure it, is a groundbreaking concept in pain control because it specifically addresses pain outcomes. The current study tested the feasibility of the PRS in pain patients who live in a collectivist social framework deeply influenced by Confucianism and Buddhism and discovered factors that cannot be explained solely with the original PRS. Although PR is highly correlated with engagement/disengagement coping methods in this study, qualitative data still showed that there are factors beyond individual control that significantly impact coping experiences. A commonality among the three themes found in the interviews is the feeling of helplessness. This affective experience can result from a learned experience that no matter how much one tries, there is no instant cure for pain [68]. Narrative data showed that most participants were still searching endlessly for an answer. However, the choice of coping methods varies among individuals with different levels of PR. The motivational tenacity construct in PR may lead individuals with higher PR to actively participate in engagement coping despite feelings of helplessness. A potential implication to the clinical field would be to adopt a shared decision-making process to decide on a culturally acceptable treatment objective for the individual and then assess the individual’s level of PR to decide on the intensity of the rehabilitation program.

The limitations of this study include the relatively small sample size in the validation study. According to previous studies, sample size can lead to dangerous interpretations of various goodness-of-fit indices when evaluating a model, because some indices are very sensitive to sample size, such as Chi-square and the RMSEA [56]. This is why articles suggest reporting several fitness indices. Another limitation is that individuals who fit certain characteristics were unable to participate in the interviews. The recruitment of this study took place in the pain clinic of a medical center, and some individuals may not have access to medical resources due to distance, mobility, or financial reasons, which limits the recruitment of potential participants. Furthermore, the difficulty in recruitment may reflect the cultural norm in which suppression is highly valued [62], making people less willing to talk about their experiences of pain. The human resources involved in this study could be a factor that limits this study to a single medical center. Although collaborations between clinicians from different medical centers might be a potential solution to increase recruitment sources, the scarcity of talent who can assist research tasks such as arranging participants, accounting, and completing a huge amount of paperwork can still be challenging.

## 5. Conclusions

The validated PRS-C showed appropriate psychometric properties. Using quantitative questionnaires and qualitative interviews, the current study demonstrated that individuals with higher PR displayed different coping styles from individuals with lower PR. Qualitative data also found that conformity to socially desirable behaviors (i.e., “People said”), is a part of pain coping in individual living in this collectivist-oriented area. This study suggests that items related to interpersonal relations and items related to spiritual needs should be added to the PRS when evaluating the PR of individuals from a collectivist framework. Like the development of the WHOQOL [30,31], future studies should work on the development of a PRS that includes culturally sensitive items. The original PRS was developed in a cultural context of individualism and measures an individual’s attitudes and behaviors toward pain. Additionally, clinical treatment programs should try to incorporate the training of PR while taking into account the influence of social norms.

## Figures and Tables

**Table 1:** Demographic characteristics of the participants for the validation study.

	Participants
Sex	Male, 13; female, 33
Age (years)	38.90 ± 11.51
Education (years)	14.59 ± 2.72
Social welfare benefits	Yes, 5; no, 41
Smoking	Smokers, 9; non-smokers, 37
Employment status	Unemployed, 7; part-time employed, 4; full-time employed, 31; retired, 4
Psychiatric disorder	Yes, 15; no, 31
Sleep disturbances	Yes, 21; no, 25
PRS-C	
14-item	36.33 ± 13.06
10-item	26.63 ± 9.11
PHQ-9	12.02 ± 7.33
WHOQOL-BREF	96.13 ± 20.05
BRS	17.85 ± 4.54
PCS	23.54 ± 13.08

PRS-C: Chinese version of the Pain Resilience Scale; PHQ-9: Patient Health Questionnaire (9 items); WHOQOL-BREF: WHO Quality of Life-BREF Taiwan version; BRS: Brief Resilience Scale; PCS: Pain Catastrophizing Scale.

**Table 2:** Item translations/original text and factor loadings from confirmatory factor analysis.

PRS-C		14-Item (Original)		10-Item (Final)	
Item	當我面對強烈或持續的疼痛時…/when faced with intense or prolonged pain…	Factor 1	Factor 2	Factor 1	Factor 2
1	我會振作起來/Get back out there	0.99	–0.34		0.91
2	我仍會嘗試實現我的目標/I still work to accomplish my goals	0.84			0.80
3	我會設法通過障礙/I push through it	0.97			0.84
4	我會試著保持工作/I try to continue working	0.57			0.51
5	我想要保持活躍的生活/I like to stay active	0.64			removed
6	我專注於積極的想法/I focus on positive thoughts	0.54	0.43	0.64	0.31
7	我保持積極的態度/I keep a positive attitude	0.51	0.46	0.70	
8	這並不影響我的幸福/It doesn’t affect my happiness	0.60		removed	
9	我仍可以在生活中找到快樂/I still find joy in my life	0.75		removed	
10	我保持有希望的態度/I keep a hopeful attitude	0.60	0.38	0.76	
11	我不讓它擊垮我/I don’t let it get me down	0.31	0.55	0.84	
12	我不讓它煩擾我/I don’t let it upset me	0.31	0.65	0.83	
13	我避免消極的想法/I avoid negative thoughts		1.04	0.93	
14	我試著保持放鬆/I try to stay relaxed		1.00	removed	
Proportion explained		0.64	0.36	0.58	0.42
Total variance		0.71		0.72	

PRS-C: Chinese version of the Pain Resilience Scale. Original text adapted from Slepian et al. (2016) [1]. Permission to validate was granted by the original author of the PRS.

**Table 3:** Model fitness indices (95% CI).

Statistics	14-Item (Original)	10-Item (You and Jackson, 2021 [5])	10-Item (Final)
Cronbach’s α	0.96	0.96	0.95
Subscale-BP	0.89	0.87	0.87
Subscale-CAP	0.95	0.95	0.94
χ^2^ (df)	202.99 (76)	62.72 (34)	44.08 (34)
*p*-value	<0.001	0.002	0.115
RMSEA (90% CI)	0.19 (0.16, 0.22)	0.14 (0.08, 0.19)	0.08 (0.00, 0.14)
SRMR	0.08	0.05	0.05
CFI	0.81	0.94	0.97
TLI	0.77	0.92	0.97
AIC	1496.10	1061.01	1042.92
BIC	1549.13	1099.41	1081.32

Subscale-BP: Behavioral Perseverance subscale including items PRS-C1 to PRS-C4; Subscale-CAP: Cognitive/Affective Positivity subscale including items PRSC-6, PRSC-7, and PRSC-10 to PRSC-13; RMSEA: root mean square error of approximation; SRMR: standardized root mean square residual; CFI: comparative fit index; TLI: Tucker–Lewis index; AIC: Akaike information criterion; BIC: Bayesian information criterion; CI: confidence interval.

**Table 4:** Demographic classifications of interviewees (PRS-C scores in parentheses).

		Religious Belief		No Religious Belief		Total
		Social Welfare	No Social Welfare	Social Welfare	No Social Welfare	
Male	Opioids	B2 (2) B6 (10) B17 (22)	B9 (12) B16 (21) B20 (21)	B21 (6)		7
	No Opioids	B19 (27)	B7 (28) B10 (17)		B1 (38) B5 (24) B11 (31)	6
Female	Opioids	B4 (38) B8 (12) B22 (19)	B13 (8) B14 (10) B23 (5)		B3 (17)	7
	No Opioids	B18 (35)	B12 (35) B24 (20)		B15 (25)	4
Total		8	10	1	5	24

P + number indicates participant number.

**Table 5:** Demographic characteristics of the qualitative interview study.

	Participants
Sex	Male, 12; female, 12
Age (years)	42.38 ± 12.48
Education (years)	13.29 ± 2.47
Social welfare benefits	Disability, 5; financial, 4; both, 2; none, 13
Employment status	Unemployed, 11; part-time employed, 4; full-time employed, 8; retired, 1
Marital status	Married, 5; divorced, 6; single, 12; widowed, 1
Prescription opioid	Yes, 16; no, 8
Religion	Buddhism, 4; Catholic, 2; Christian, 1; Tao, 13; none, 4
PRS-C	20.13 ± 10.44

PRS-C: Chinese version of the Pain Resilience Scale; PHQ-9: Patient Health Questionnaire (9 items); WHOQOL-BREF: WHO Quality of Life-BREF Taiwan version; BRS: Brief Resilience Scale; PCS: Pain Catastrophizing Scale.

## Data Availability

Raw data and R analytical codes can be found in the Supplementary Files.

## Supplementary Materials

The following supporting information can be downloaded at: https://www.mdpi.com/article/10.3390/healthcare12191979/s1, list S1: Interview questions; Table S1: Item descriptive statistics and exploratory indices.; analytical code S1: validation3; raw data excel file: validation. Figure 1 and results of Table 3 can be generated by the R codes in the Supplementary File.
