# Supplementary material for: Pain Resilience and Coping Behaviors in Individuals in a Collectivist Social Context
Source: Healthcare (Basel). 2024 Oct 4;12(19):1979. doi: 10.3390/healthcare12191979 (PMC11477074; doi:10.3390/healthcare12191979)
Supplement: Supplementary file 1 [file healthcare-12-01979-s001.zip › interview questions.pdf]

1. 請問您的疼痛狀況多久了/How long have you been in pain?
2. 疼痛開始以來，你面對疼痛的方式有什麼變化？例如一開始疼痛時你做過哪些努力去改善，到後來你又怎麼做？/Since your pain started, are there any changes in your ways of coping with pain? For instance, how did you initially cope with pain, and then how did you cope with it later on?
3. 你曾經做過哪些治療？/What treatments have you received?
4. 現在還有在做那些治療？/What are the treatments you still receive?
5. 你覺得這個疼痛對你的人生或是生涯規劃有什麼影響？/Any changes in life plans due to this pain?
6. 你現在都怎麼度過每一天？/How do you spend your days now?
7. 疼痛對你的生活最大的影響或是不便利是什麼？或是疼痛對你的生活帶來什麼問題？/How does this pain affect your daily life? Is it causing trouble for your daily living?
8. 每當你感受到疼痛好像又要發作時，你會有什麼念頭以及會做什麼？/Your first thoughts and response when you feel that the pain is going to attack.
9. 除了吃藥以外，你還會做什麼事情來想辦法改善你的疼痛？/In addition to medications, what other things do you do to alleviate pain?
10. 疼痛以前你怎麼看待你自己？現在又怎麼看待自己？/How did you regard yourself before you had pain? And now?
11. 那你身邊的人怎麼跟你相處的？他們會給你任何止痛或是治療的建議嗎？還是你會主動找人討論？都可以找誰討論？/How do you get along with people around you? Do you receive any advice from them? Or do you actively seek advice from other people (to alleviate pain)? Who do you talk to?
12. 疼痛前、後人際關係有什麼變化嗎？/Any changes in interpersonal relationships since the pain started?

13. 你曾經求助宗教或是其他非醫療的方法試圖去改善疼痛嗎？/Have you tried to seek help from religion or other non-medical sources?
14. 呈上，若有的話，是什麼情況？/If yes, tell me more about it.
15. 有沒有什麼事情或興趣是你以前很喜歡的但是因為疼痛所以需要放棄？/Is there anything that you used to enjoy, but you had to give up because of pain?
16. 很多醫生應該跟你說過疼痛很可能就會這樣跟著你一輩子，你自己對於這個說法，你怎麼想？/You might have heard from many doctors that chronic pain might persist for the rest of your life. What do you think?
17. 對於未來你有什麼想法或抱持什麼希望？/What are your hopes for the future?

今天我大致聽到了你的面對疼痛歷程的經驗是_(簡略的概述受訪者剛才所說的答案)_，有沒有記錯的地方？/I have heard (brief recap of the interview). Did I get everything right? Is there anything else you would like to share on this topic?
