# Supplementary material for: Pain Resilience and Coping Behaviors in Individuals in a Collectivist Social Context
Source: Healthcare (Basel). 2024 Oct 4;12(19):1979. doi: 10.3390/healthcare12191979 (PMC11477074; doi:10.3390/healthcare12191979)
Supplement: Supplementary file 1 [file healthcare-12-01979-s001.zip › Supplementary table.pdf]

Supplementary Table. Item descriptive statistics and exploratory indices.

Item number	Item mean (sd)	corrected item-total correlation	Cronbach's α
1	2.74 (1.08)	0.61	0.96
2	2.57 (1.13)	0.72	0.96
3	2.85 (1.01)	0.77	0.95
4	2.98 (0.91)	0.66	0.96
5	2.46 (1.11)	0.82	0.95
6	2.41 (1.24)	0.88	0.95
7	2.63 (1.61)	0.88	0.95
8	2.26 (1.48)	0.78	0.96
9	2.5 (1.26)	0.85	0.95
10	2.59 (1.17)	0.89	0.95
11	2.87 (1.15)	0.77	0.95
12	2.46 (1.22)	0.85	0.95
13	2.54 (1.11)	0.66	0.96
14	2.48 (1.13)	0.64	0.96
Kaiser-Meyer-Olkin factor adequacy		0.86	
Intraclass correlation		0.60	
Cronbach's α		0.96	
Total variance		0.71	

sd: standard deviation
